# Hexokinase 2 promoted cell motility and proliferation by activating Akt1/p-Akt1 in human ovarian cancer cells

**DOI:** 10.1186/s13048-022-01027-8

**Published:** 2022-08-11

**Authors:** Xueye Tian, Dan Liu, Xiaohang Zuo, Xiaoli Sun, Mengmin Wu, Xu Li, Yue Teng

**Affiliations:** 1 Department of Obstetrics and Gynecology/Centre for Translational Medicine, the First Affiliated Hospital of Xi’an Jiaotong University, Xi’an, 710061 China; 2 Department of Endocrinology, Xijing 986 Hospital, Fourth Military Medical University, Xi’an, 710032 China; 3 Department of Pathology, Baoji Maternal and Child Health Hospital, Baoji, 721099 China; 4 Centre for Translational Medicine, The First Affiliated Hospital of Xi’an Jiaotong University, Xi’an, 710061 China

**Keywords:** HK2, EMT, Akt1, Ovarian cancer, Proliferation

## Abstract

**Background:**

Recently, increasing evidence has indicated that elevation of Hexokinase 2 (HK2) plays an important role in several cancers on regulating cell motility and growth. However, its role on regulating cell EMT in human ovarian cancer still less to known.

**Methods:**

The transwell and wound-healing assay were used to detect the effective of HK2 on regulating motility of ovarian cancer cells. Real Time PCR and Western Blotting were used to explore the changing of EMT-related proteins in HK2-modified cells. The clonogenic formation, cell growth curves and MTT assays were used to evaluate the effective of HK2 on regulating cell proliferation in HK2-modified cells. The flow cytometry was used to detect the differences in the distribution of cells in the cell cycle between the HK2-modified cells and their control cells. The correlation of HK2 and Akt1/p-Akt1 was explored by using Western Blotting, Akt1 inhibitor (MK2206) and transient transfection of an Akt1 recombinant plasmid. The potential correlation between HK2 and EMT-related proteins in human ovarian cancer tissues and OV (ovarian serous cystadenocarcinoma) was confirmed by using Pearson correlation analysis and TIMER 2.0.

**Results:**

In ovarian cancer cells, overexpressing of HK2 enhanced cell motility by inducing of EMT-related proteins, such as CDH2, fibronectin, MMP9, ZEB1, ZEB2 and vimentin. Moreover, overexpressing of HK2 promoted cell growth by reducing p21 and p27 expression in ovarian cancer cells. Further studies demonstrated that this promotion of cell motility and growth by HK2 was probably a result of it activating of Akt1 (p-Akt1) in ovarian cancer cells. Additionally, the positive correlation between HK2 and p-Akt1, fibronectin, MMP9 expression in human ovarian cancer samples was verified by using Pearson correlation analysis. The positive correlation between HK2 and CDH2, fibronectin, MMP9, ZEB1, ZEB2 and vimentin in OV (ovarian serous cystadenocarcinoma) was confirmed by using TIMER 2.0.

**Conclusion:**

This study demonstrated that HK2 could induce EMT-related proteins and reduce cell cycle inhibitor by activating Akt1 in human ovarian cancer cells, subsequently enhancing cell motility and growth, suggesting that HK2 participate in the malignant process of ovarian cancer by interacting with Akt1.

## Background

Ovarian cancer is the second most common cause of gynecological cancer-related deaths in women worldwide. Epithelial ovarian cancer (EOC) is the main pathological subtype, it is still the most lethal gynecological cancer, accounting for more than 95% of ovarian malignancies. Although epithelial ovarian cancers (EOCs) are sensitive to chemotherapy, however, patients frequently develop recurrent disease following initial platinum-taxane chemotherapy [1–3]. Tumor metastasis is closely associated with poor prognosis and is also the main cause of death in patients with ovarian cancer. Metastasis is the main cause of poor prognosis in patients with ovarian cancer [4]. Because there are no specific symptoms at the early stage of ovarian cancer, most patients are diagnosed at stage III/IV when the tumor has metastasized throughout the peritoneal cavity [5]. At present, the molecular mechanisms that promote the invasion and metastasis of ovarian cancer cells remain poorly characterized.

The phosphoinositide 3-kinase (PI3K)–AKT pathway is the most commonly activated pathway in human cancers. Oncogenic activation of the PI3K–AKT pathway in cancer cells reprogrammes cellular metabolism by augmenting the activity of nutrient transporters and metabolic enzymes, thereby supporting the anabolic demands of aberrantly growing cells. Activation of PI3K at the plasma membrane stimulates phosphorylation of its downstream effectors to produce the second messenger. AKT (also known as protein kinase B or PKB), is one of the major downstream effectors of PI3K–AKT pathway. Active AKT phosphorylates a large and diverse array of down-stream substrates, further influencing a variety of cell biological functions, including cell growth, proliferation, survival and EMT. For instance, Active AKT promoted cell proliferation and EMT in endometrial cancer [6], colorectal cancer [7], breast cancer [8], osteosarcoma [9], further promoting tumorigenicity and metastasis, also including ovarian cancer [10–13],

Hexokinase 2 (HK2) is the most active isozyme of the hexokinase family, is an essential regulator of glycolysis. HK2 induction in most in cancer cells couples metabolic and proliferative activities, and its genetic ablation inhibits malignant growth in mouse models [14, 15]. HK2 is mainly expressed in insulin-sensitive tissues and predominant in malignant or rapidly proliferating tumors (such as cervical cancer, breast cancer, lung cancer, prostate cancer, hepatocyte cell cancer), deleting HK2 inhibits tumor progression with no sign of adverse physiological effects [15–17]. In ovarian cancer, elevated HK2 expression was significant associated with chemoresistance, suggesting that HK2 might contribute to cancer progression [18]. However, the potential function of HK2 on regulating cell proliferation and motility in human ovarian cancer cells was still less to known. In this study, elevation of HK2 in human ovarian cancer cells significantly promoted cell growth and motility capacity, and further study implied that active of Akt1 in HK2-modified cells must associated with the enhanced cell proliferation and motility.

## Results

### HK2 promotes cell migration and invasion of human ovarian cancer cells in vitro

**Fig. 1:**
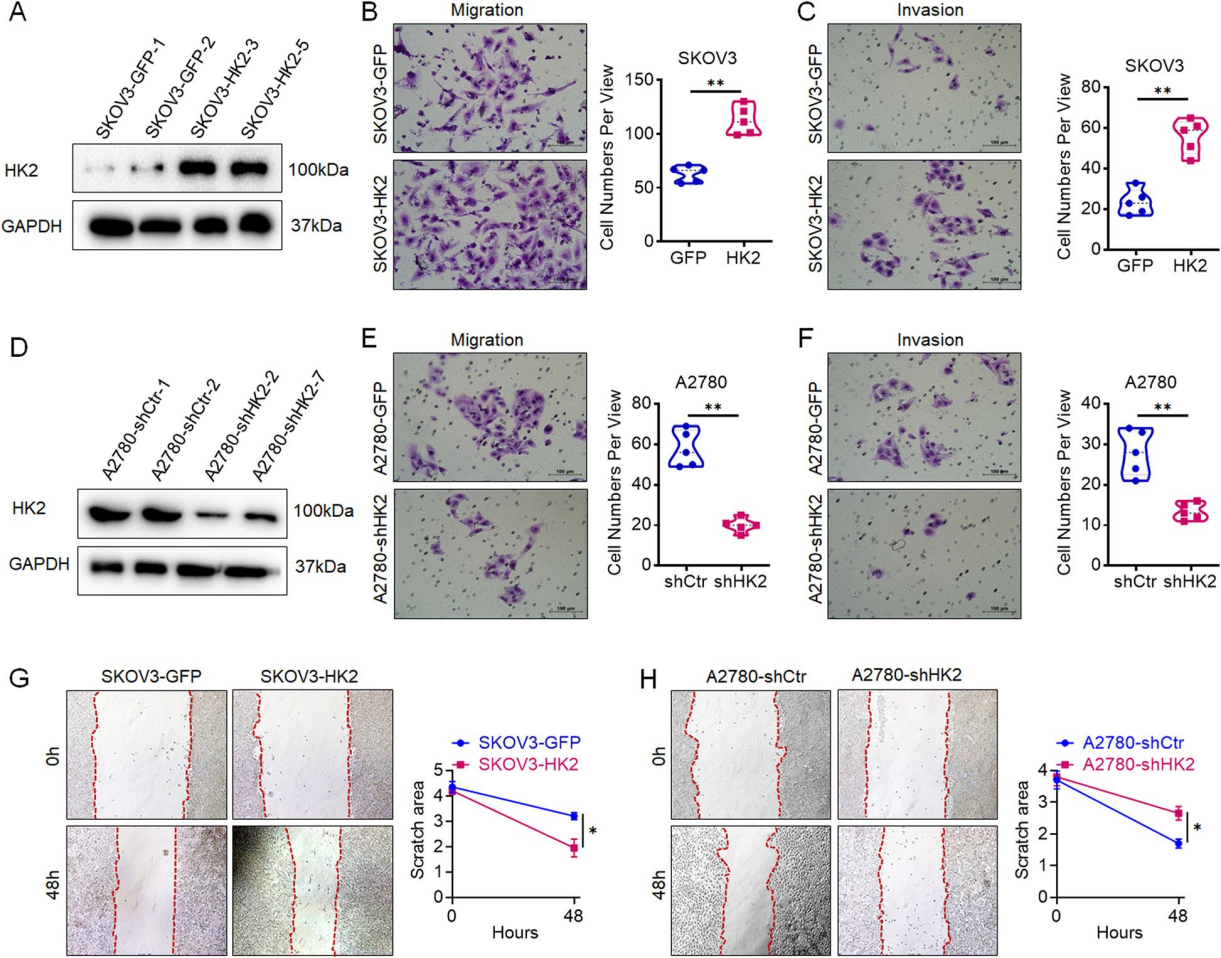
HK2 enhances the migration and invasion ability in human ovarian cancer cells in vitro. Stably transfected cell lines were identified by western blotting: (**A**) SKOV3-GFP and SKOV3-HK2; (**D**) A2780-shCtr and A2780-shHK2. The migratory capacities were analyzed by the transwell assay, and the number of migratory cells is shown (scale bar, 100 μm): (**B**) SKOV3-GFP and SKOV3-HK2; (**E**) A2780-shCtr and A2780-shHK2. The invasive capacities were analyzed by the transwell assay, and the number of migratory cells is shown (scale bar, 100 μm): (**C**) SKOV3-GFP and SKOV3-HK2; (**F**) A2780-shCtr and A2780-shHK2. The migratory potential was analyzed by wound-healing assays performed for 0 and 48 h: (**G**) SKOV3-GFP and SKOV3-HK2; (**H**) A2780-shCtr and A2780-shHK2. The data are shown as the mean ± SD of three independent experiments. * p < 0.05, ** p < 0.01 vs. control using one-way ANOVA

To further investigate the function of HK2 on regulating migratory capacity in human ovarian cancer cells, exogenous HK2 was stably overexpressed in SKOV3 (SKOV3-GFP and SKOV3-HK2, Fig. 1A) cells; conversely, endogenous expression of HK2 was knocked down by stably transfecting shRNA plasmids in A2780 (A2780-shCtr and A2780-shHK2, Fig. 1D) cells. Firstly, transwell assays was performed to evaluate the capacity for cell motility in HK2-modified human ovarian cancer cells. As shown in Fig. 1B, the transwell migration analysis revealed that the number of SKOV3-HK2 cells that migrated across the membrane was much more than SKOV3-GFP cell (112.40 ± 13.18 vs 62.80 ± 7.39, *p* < 0.05, Fig. 1B). Conversely, the number of A2780-shHK2 cells that migrated across the membrane was much less than A2780-shCtr cells (20.40 ± 3.61 vs 57.80 ± 8.93, *p* < 0.05, Fig. 1E). Furthermore, the transwell invasive analysis revealed that the number of SKOV3-HK2 cells that migrated across the membrane was much more than SKOV3-GFP cell (56.00 ± 8.42 vs 23.60 ± 6.31, *p* < 0.05, Fig. 1C). Conversely, the number of A2780-shHK2 cells that migrated across the membrane was much less than A2780-shCtr cells (13.40 ± 2.07 vs 28.00 ± 5.62, *p* < 0.05, Fig. 1F).

Additionally, wound-healing assays revealed that a significant increase in wound closure was observed in SKOV3-HK2 cells, comparing with that observed in the SKOV3-GFP cells (*p* < 0.05, Fig. 1G). Conversely, a significant decrease in wound closure was found in A2780-shHK2 cells, comparing with that observed in the A2780-shCtr cells (*p* < 0.05, Fig. 1H). All of these results demonstrate that exogenously expressed HK2 in human ovarian cancer cells significantly enhances cell migratory and invasive capacity in vitro.

### HK2 altered the expression of EMT-related proteins in human ovarian cancer cells

To explore the potential EMT-related proteins that are probably mediated by HK2 in human ovarian cancer cells to enhance cell migratory and invasive, real-time PCR and western blotting were applied to verify the expression of key EMT-related proteins in SKOV3-GFP, SKOV3-HK2, A2780-shCtr and A2780-shHK2 cells.

**Fig. 2:**
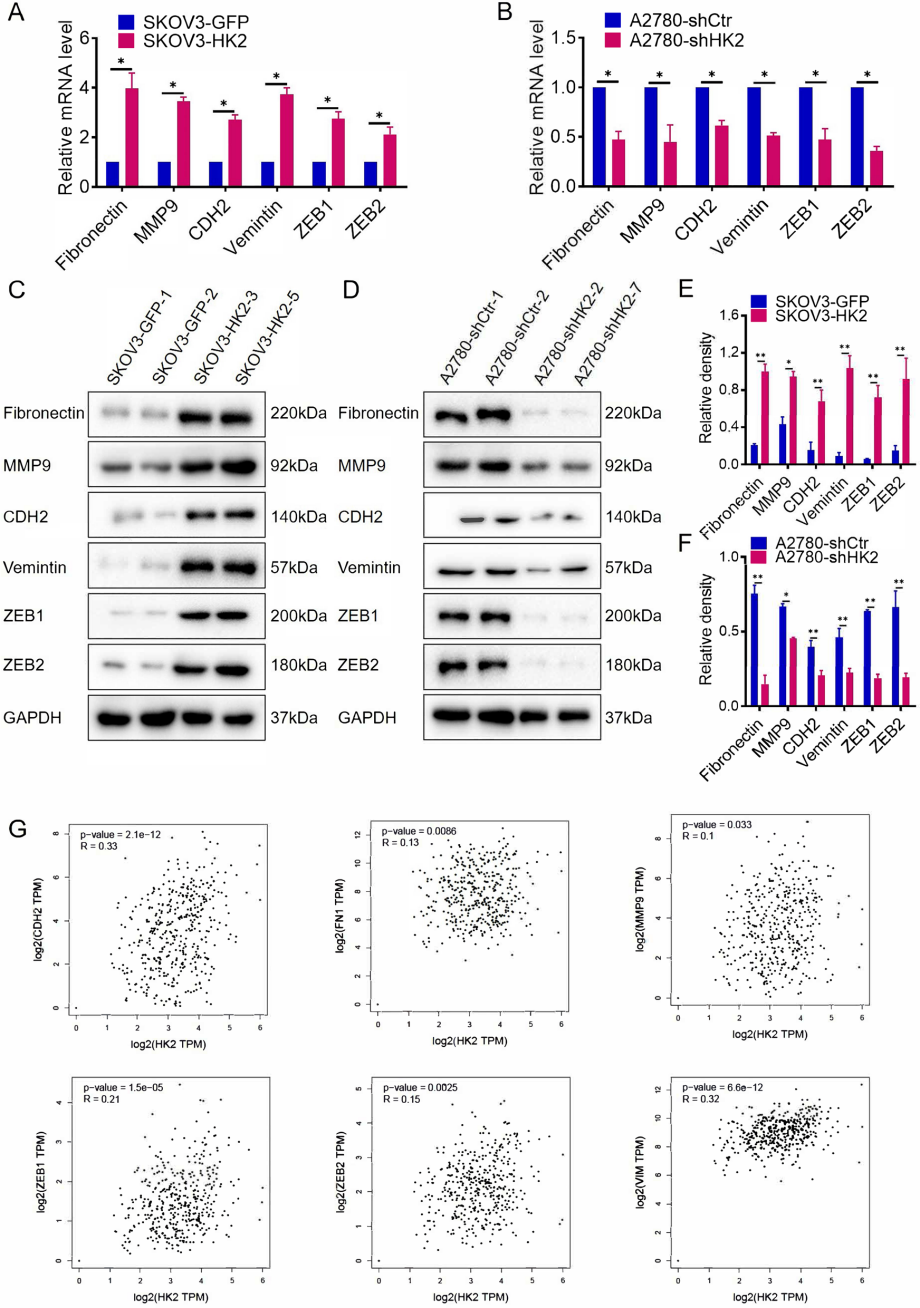
HK2 elevated the expression of EMT-related proteins in human ovarian cancer cells. The mRNA expression of Fibronectin, MMP9, CDH2, Vemintin, ZEB1, ZEB2 was detected by real-time quantitative PCR, and the quantitative analysis is shown: (**A**) SKOV3-GFP and SKOV3-HK2; (**B**) A2780-shCtr and A2780-shHK2. The protein level of Fibronectin, MMP9, CDH2, Vemintin, ZEB1, ZEB2 was detected by western blot: (**C**) SKOV3-GFP and SKOV3-HK2; (**D**) A2780-shCtr and A2780-shHK2, and the quantitative analysis is shown: (**E**) SKOV3-GFP and SKOV3-HK2; (**F**) A2780-shCtr and A2780-shHK2. **G** The positive correlation between HK2 and Fibronectin, MMP9, CDH2, Vemintin, ZEB1, ZEB2 expression in ovarian serous cystadenocarcinoma were confirmed from the GEPIA online database. The data are shown as the mean ± SD of three independent experiments. * p < 0.05, ** p < 0.01 vs. control using one-way ANOVA

As shown in Fig. 2A, the mRNA levels of Fibronectin, MMP9, CHD2, vimentin, ZEB1 and ZEB2 were much higher in SKOV3-HK2 cells than that in SKOV3-GFP cells. Conversely, the decreased mRNA levels of Fibronectin, MMP9, CHD2, vimentin, ZEB1 and ZEB2 were observed in A2780-shHK2 cells, comparing with A2780-shCtr cells (Fig. 2B, *p* < 0.05). Consistently, the protein levels of fibronectin, MMP9, CHD2, vimentin, ZEB1 and ZEB2 were much higher in SKOV3-HK2 cells than that in SKOV3-GFP cells (Fig. 2C, *p* < 0.05). And the decreased protein levels of Fibronectin, MMP9, CHD2, vimentin, ZEB1 and ZEB2 were observed in A2780-shHK2 cells, comparing with A2780-shCtr cells (Fig. 2D, *p* < 0.05).

Additionally, the positive correlation between HK2 and fibronectin, MMP9, CHD2, Vimentin, ZEB1 and ZEB2 in human ovarian cancer were confirmed from the GEPIA online database (Fig. 4G, *p* < 0.05). These results suggested that the enhanced cell migratory and invasive capacity that mediated by HK2 likely depends on the up-regulated EMT-related proteins expression in human ovarian cancer.

### HK2 promotes cell growth by reducing p21/p27 expression in human ovarian cancer cells

**Fig. 3:**
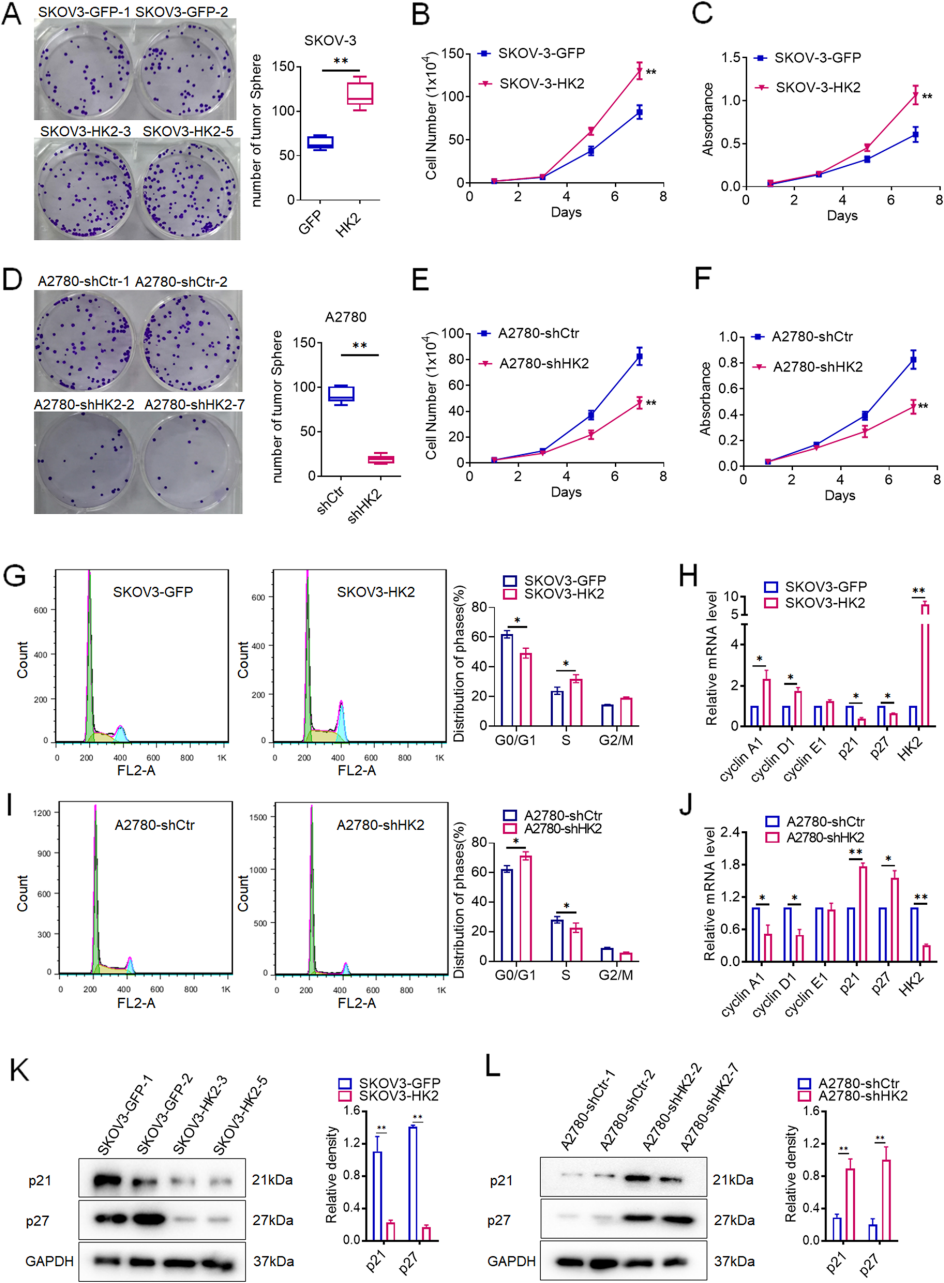
HK2 promoted cell proliferation of human ovarian cancer cell lines in vitro. The colony formation assay was used to detected the long-term cell survival and growth in HK2-modified cells and control cells and the quantitative analysis is shown: (**A**) SKOV3-GFP and SKOV3-HK2; (**D**) A2780-shCtr and A2780-shHK2. The growth curves were used to detected the cell proliferation in HK2 modified cells: (**B**) SKOV3-GFP and SKOV3-HK2; (**E**) A2780-shCtr and A2780-shHK2. The MTT assay were used to detected the cell viability in HK2 modified cells: (**C**) SKOV3-GFP and SKOV3-HK2; (**F**) A2780-shCtr and A2780-shHK2. The cell cycle was analyzed in HK2 modified cells by using flow cytometry: (**G**) SKOV3-GFP and SKOV3-HK2; (**I**) A2780-shCtr and A2780-shHK2. The mRNA expression of cyclin A1, cyclin D1, cyclin E1, p21, p27 and HK2was detected by real-time quantitative PCR, and the quantitative analysis is shown: (**H**) SKOV3-GFP and SKOV3-HK2; (**J**) A2780-shCtr and A2780-shHK2. The protein level of p21 and p27 was detected by western blot and the quantitative analysis is shown: (**K**) SKOV3-GFP and SKOV3-HK2; (**L**) A2780-shCtr and A2780-shHK2

In this study, the clonogenic formation assay was used to detected the potential function of HK2 on regulating cell proliferation in human ovarian cancer cells. As shown in Fig. 3, the number of cell clones in SKOV3-HK2 cells was much more than SKOV3-GFP cell (117.70 ± 14.14 vs 63.67 ± 6.80, *p* < 0.05, Fig. 3A). Conversely, the number of cell clones in A2780-shHK2 cells was much less than A2780-shCtr cells (19.33 ± 4.46 vs 91.00 ± 8.75, *p* < 0.05, Fig. 3D). Moreover, the cell growth curves and MTT assays revealed that the SKOV3-HK2 cells grew much faster than SKOV3-GFP cells (Fig. 3B and C, *p* < 0.05). Conversely, when endogenous expression of HK2 was knocked down in A2870-shHK2 cells, cells grew much slower than A2780-shCtr cells (Fig. 3E and F, *p* < 0.05). These results demonstrated that HK2 enhanced cell growth in human ovarian cancer cells.

Moreover, the flow cytometry was used to detect the differences in the distribution of cells in the cell cycle between the HK2-modified cells and their control cells. As shown in Fig. 3G, the decreased proportion of cells in the G0/G1 phase (49.11 ± 3.34) and increased proportion of cells in the S phase (32.02 ± 2.68) was observed in in SKOV3-HK2 cells, comparing with SKOV3-GFP cells (G0/G1 phase: 61.91 ± 2.49; S phase: 23.83 ± 2.45). Conversely, the increased proportion of cells in the G0/G1 phase (71.39 ± 2.72) and decreased proportion of cells in the S phase (22.75 ± 3.19) was observed in in A2870-shHK2 cells, comparing with A2780-shCtr cells (G0/G1 phase: 62.58 ± 2.23; S phase: 28.14 ± 2.23, Fig. 3I). These results suggested that HK2 could accelerate cell cycle progression in human ovarian cancer cells.

Additionally, real-time PCR was used to verify the expression of key cell cycle-related proteins in SKOV3-GFP, SKOV3-HK2, A2780-shCtr and A2780-shHK2 cells. As shown in Figs. 3H, the mRNA levels of cyclin A1, cyclin D1 and cyclin E1 were much higher, p21 and p27 were much lower in SKOV3-HK2 cells than that in SKOV3-GFP cells. Conversely, the mRNA levels of cyclin A1, cyclin D1 and cyclin E1 were much lower, p21 and p27 were much higher in A2780-shHK2 cells, comparing with A2780-shCtr cells (Fig. 3J, *p* < 0.05). Consistently, the decreased protein levels of p21 and p27 were observed in HK2 overexpressed SKOV3 cells (Fig. 3K, *p* < 0.05), and the increased protein levels of p21 and p27 were observed in HK2 knocked down A2780 cells (Fig. 3L, *p* < 0.05).

### HK2 elevated Akt1 and p-Akt1 expression in human ovarian cancer cells

**Fig. 4:**
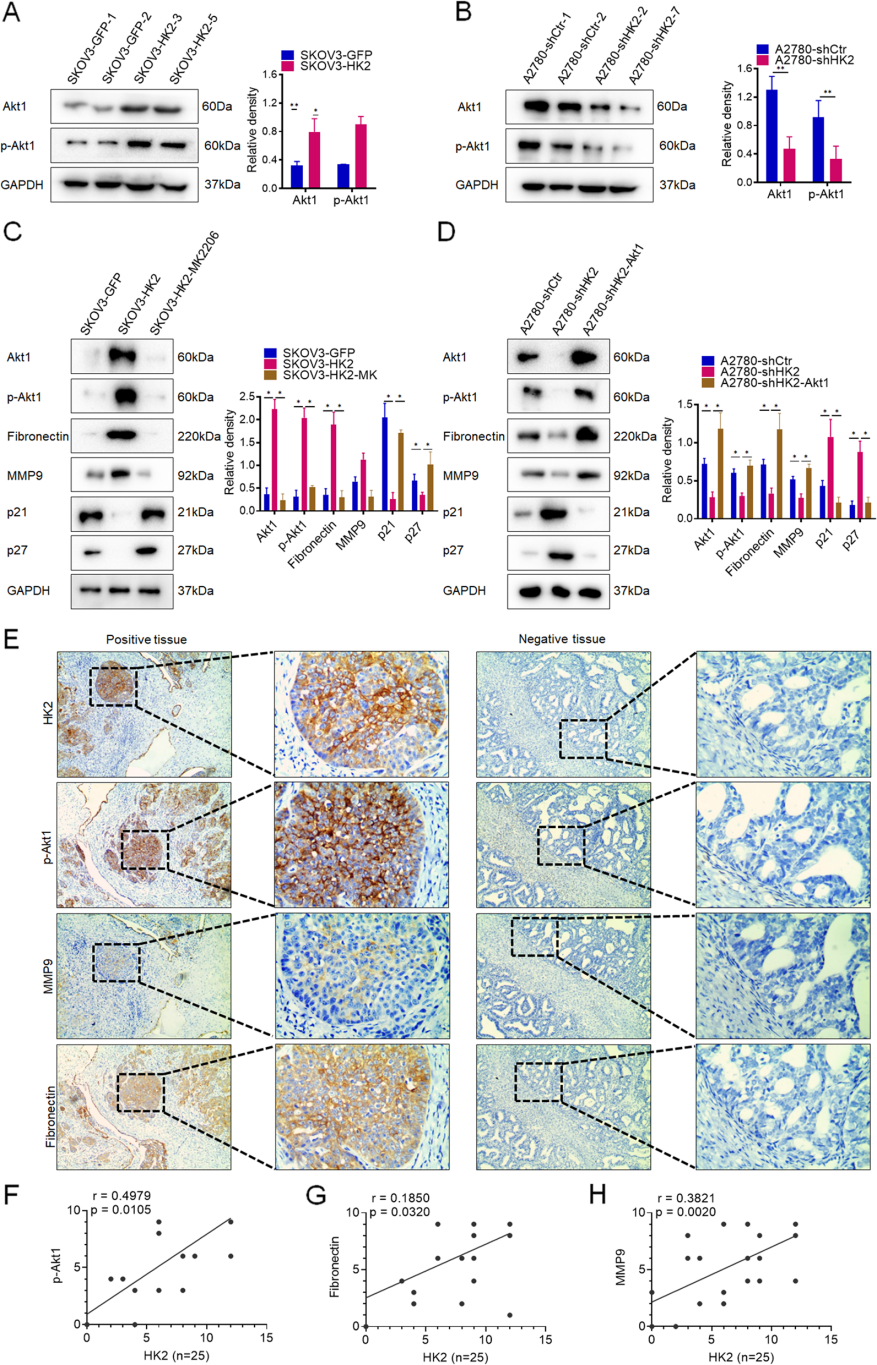
HK2 elevated Akt1 and p-Akt1 expression in human ovarian cancer cells. The protein level of Akt1 and p-Akt1 was detected by western blot and the quantitative analysis is shown: (**A**) SKOV3-GFP and SKOV3-HK2; (**B**) A2780-shCtr and A2780-shHK2. **C** The protein level of Akt1, p-Akt1, Fibronectin, MMP9, p21 and p27 was detected by western blot in MK2206 treated SKOV3-HK2 cells and the quantitative analysis is shown. **D** The protein level of Akt1, p-Akt1, Fibronectin, MMP9, p21 and p27 was detected by western blot in A2780-shHK2 cells that transiently transfected with an Akt1 recombinant plasmid, and quantitative analysis is shown. **E** The expression of HK2, p-Akt1, MMP9 and FN1 was detected in serial sections of human ovarian cancer tissues by using immunocytochemistry analysis (scale bar, 50 and 10 μm). The correlation between HK2 and p-Akt1(**F**), Fibronectin (**G**), MMP9 (**H**), in human ovarian cancer tissues was confirmed by using Pearson correlation analysis, *n =* 25

Previous study demonstrated that HK2 participated in regulating Akt1 and p-Akt1 expression in various cancer. In order to determine whether the Akt1 and p-Akt1 expression could be regulated under HK2 expression in human ovarian cancer cell lines, western blot was used to detected the protein levels of Akt1 and p-Akt1 in HK2- modified human ovarian cancer cells. As shown in Fig. 4, both of the protein level of Akt1 and p-Akt1 were increased in HK2 overexpressed SKOV3 cells (Fig. 4A, *p* < 0.05). Conversely, the reduction of protein level of Akt1 and p-Akt1 were observed in HK2 knocked down A2780 cells (Fig. 4B, *p* < 0.05).

Furthermore, in order to determine whether the alteration of Akt1 and p-Akt1 expression under HK2-modified was responsible for the changing of cell growth, migratory and invasive capacity in human ovarian cancer cells, MK2206, an inhibitor of Akt1/p-Akt1, was used to block the elevated expression of Akt1 and p-Akt1 in HK2 overexpressed SKOV3 cells. As shown in Fig. 4C, when the elevated expression of Akt1 and p-Akt1 in SKOV3-HK2 cells were blocked by MK2206, reduced Akt1, p-Akt1, fibronectin, MMP9 and induced p21, p27 expression was observed in MK2206-treated SKOV3-HK2 (Fig. 4C, *p* < 0.05). Moreover, the protein level of Akt1 and p-Akt1 expression was rescued in HK2-knockdown A2780-shHK2 cells via transient transfection of an Akt1 recombinant plasmid (pIRES2-AcGFP-Akt1). As shown in Fig. 4D, an induced Akt1, p-Akt1, fibronectin, MMP9 and reduced p21, p27 expression was observed in A2780-shHK2-Akt1 cells. These results demonstrated that HK2 could elevate Akt1 and p-Akt1 expression in human ovarian cancer cells, subsequently enhancing cell motility by inducing Fibronectin and MMP9 expression, promoting cell growth by reducing p21 and p27 expression.

Moreover, in order to confirm the positive correlation between the expression of HK2 and p-Akt1, Fibronectin and MMP9 in human ovarian cancer tissues, serial sections of human ovarian cancer tissues (*n =* 25) were immunostained with antibodies specific for HK2, p-Akt1, fibronectin and MMP9 (Fig. 4E). As shown in Fig. 4, the positive correlation between HK2 and p-Akt1 (Fig. 4F, *r =* 0.4979, *p* = 0.0105), fibronectin (Fig. 4G, *r =* 0.1850, *p* = 0.0320) and MMP9 (Fig. 4H, *r =* 0.3821, *p* = 0.0020) in these human ovarian cancer tissues was confirmed by using Pearson correlation analysis.

**Fig. 5:**
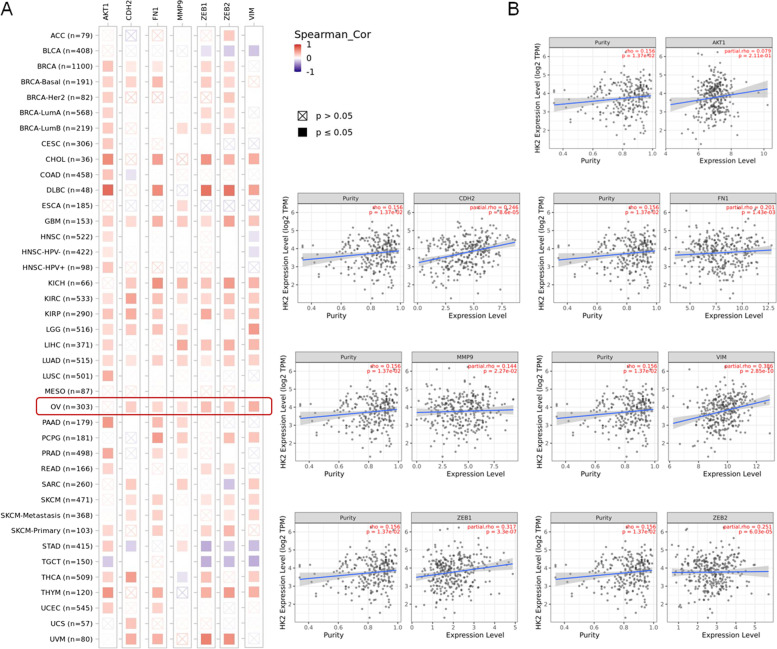
The correlation between HK2 and Akt1, CDH2, fibronectin, MMP9, ZEB1, ZEB2 and vimentin in various type of cancer. **A** The correlation between HK2 and Akt1, CDH2, fibronectin, MMP9, ZEB1, ZEB2 and vimentin in various type of cancer was confirmed by using TIMER 2.0 (http://timer.cistrome.org/). **B** The positive correlation between HK2 and CDH2, fibronectin, MMP9, ZEB1, ZEB2 and vimentin in OV (ovarian serous cystadenocarcinoma) was confirmed by using TIMER 2.0 (http://timer.cistrome.org/)

**Fig. 6:**
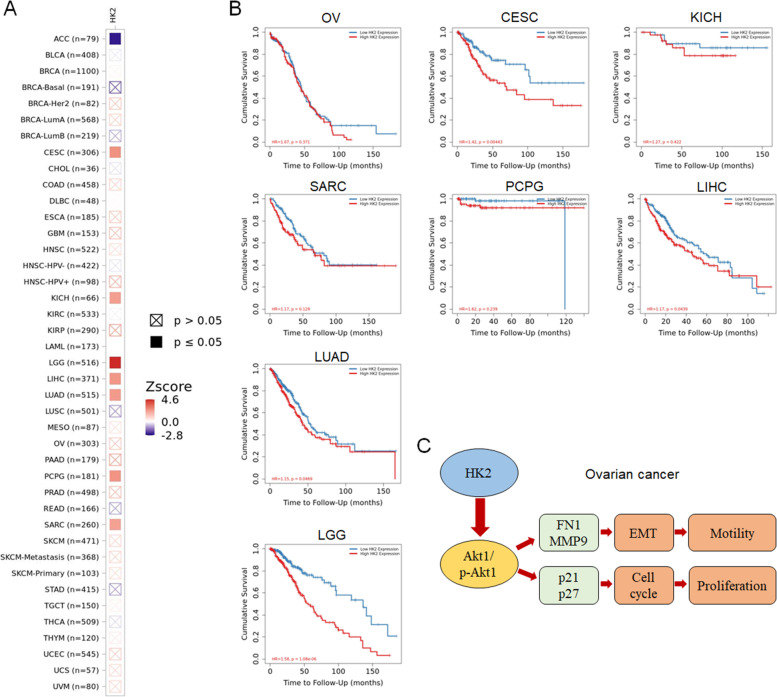
The correlation between HK2 and patient outcomes in various type of cancer. **A** The correlation between HK2 and patient outcomes in various type of cancer was confirmed by using TIMER 2.0 (http://timer.cistrome.org/). **B** The positive correlation between HK2 and patient outcomes in CESC, KICH, SARC, PCPG, LIHC, LUAD and LGG was confirmed by using TIMER 2.0 (http://timer.cistrome.org/). **C** Proposed model of the mechanisms by which HK2 promotes cell motility and growth in human ovarian cancer

Additionally, as shown in Fig. 5, the positive correlation between HK2 and CDH2, fibronectin, MMP9, ZEB1, ZEB2 and vimentin in OV (ovarian serous cystadenocarcinoma) was confirmed by using TIMER 2.0 (http://timer.cistrome.org/) [19]. Although the expression of HK2 in OV (ovarian serous cystadenocarcinoma) was not so correlation between the prognosis of patient, however, the expression of HK2 was associated with a poorly prognosis of patient in CESC (cervical squamous cell carcinoma and endocervical adenocarcinoma), KICH (kidney Chromophobe), SARC (sarcoma), PCPG (pheochromocytoma and paraganglioma), LIHC (liver hepatocellular carcinoma), LUAD (lung adenocarcinoma) and LGG (brain lower grade glioma, Fig. 6A and B, TIMER 2.0) [19].

## Discussion

Previous study had revealed that the abnormal expression of HK2 was associated with the poor prognosis in many types of tumors. In order to investigate the potential connection between HK2 and tumor prognosis in human ovarian cancer, HK2 was exogenously expressed in human ovarian cancer cell line SKOV3 and knocked down in human ovarian cancer cell line A2780, respectively. In SKOV3 cells, HK2 expression significantly enhanced cell migratory and invasive capacity. Oppositely, the capacity of cell migratory and invasive was significantly injured when HK2 was knocked down in A2780 cells. These results were consistent with the promoted function of HK2 on regulating cell migration and invasion in human ovarian cancer cells which had demonstrated in previous studies, by interacting with FAK/ERK1/2/MMP9 pathway, HK2 promoted cell migration and invasion in human ovarian cancer cells [20]. In addition, in colon cancer cells, AKT2-HK2-NF-κB/HIF1α/MMP2/MMP9 axis increased the invasion, tumorigenesis, and metastasis of in vitro and promotes lung metastasis in nude mice in vivo [21]. And HK2 also promoted cell motility and distant metastasis by elevating fibronectin/MMP2/MMP9 in cervical cancer cells [22]. In this study, further study also demonstrated that HK2 could elevate the expression of EMT-related factors in human ovarian cancer cell, like fibronectin, MMP9, CHD2, Vemintin, ZEB1 and ZEB2. Additionally, the positive correlation between HK2 and fibronectin, MMP9, CHD2, Vemintin, ZEB1 and ZEB2 in human ovarian cancer were also confirmed from the GEPIA online database. All of these results demonstrated that HK2 could enhance cell migratory and invasive capacity in human ovarian cancer cell，as well as it done in other cancer.

Moreover, HK2 was reported to be facilitate for cell proliferation and tumor formation, such as cervical cancer [23, 24], gallbladder cancer [25], diffuse large B-cell lymphoma [26], gastric cancer [27], hepatocarcinoma [28] and so on. Consistently, in this study, exogenously expressed of HK2 also promoted cell growth in human ovarian cancer cells by accelerating cell cycle progression (up-regulated the expression of cell cycle-related factors, like cyclin A1, cyclin D1 and cyclin E1).

Phosphatidylinositol-3-kinase (PI3K)/AKT/mammalian target of rapamycin (mTOR) signaling is one of the most important intracellular pathways, has been found to be dysregulated almost in all human cancers, such as breast cancer, colorectal cancer, and hematologic malignancies, which can be considered as a master regulator for cancer, which regulates cell growth, motility, survival, metabolism, and angiogenesis [29]. Akt1/p-Akt1 is one of the most important components of PI3K/AKT/mTOR pathway. By regulating EMT-related factors (like MMP2, MMP9 and fibronectin) and cell cycle suppressor (p21 and p27), Akt1/p-Akt1 involved in regulating cell motility and growth in various cancer cells. And previous studies also revealed that there had an interaction relationship between HK2 and Akt1/p-Akt1 during tumorigenesis. In this study, alteration of HK2 expression in human ovarian cancer cells affected the protein level of Akt1 and p-Akt1, suggesting that there should had a potential interaction relationship between HK2 and Akt1/p-Akt1 during human ovarian cancer progress. Moreover, when the expression of Akt1 and p-Akt1 was blocked by using an Akt1 inhibitor MK2206 in HK2 overexpressed cells or rescued by transiently transfection of an Akt1 recombinant plasmid in HK2-knockdown cells, the fibronectin, MMP9, p21and p27 also changed with it.

## Conclusion

This study demonstrated that the alteration of fibronectin, MMP9, p21 and p27 under HK2-modified must attribute to the changing of Akt1 and p-Akt1 expression, subsequently promoted cell motility and growth (Fig. 6C).

## Materials and methods

### Cell culture

Human ovarian carcinoma cell lines A2780 and SKOV3 were purchased from the American Type Culture Collection (ATCC, Rockville, MD, USA). High-glucose Dulbecco Modified Eagle Medium (DMEM, Sigma-Aldrich, St Louis, MO, USA) was used to culture A2780 and SKOV3, and 10% fetal bovine serum ((FBS; Hyclone, Thermo Scientific, Waltham, MA, USA) was added in all culture media. The overexpression and shRNA for HK2 was purchased from Gene Pharma (Shanghai, China). Lipofectamine 2000 reagent (Invitrogen, Carlsbad, CA, USA) was used to transfect the pIRES2-AcGFP-HK2 or shRNA vectors into SKOV3 and A2780 cells to generate stably transfected cell lines by treating with G418 (Calbiochem, La Jolla, CA, USA) for 3 weeks. Akt1 recombinant plasmid was stored by our laboratory. MK2206 was purchased from Selleck (Shanghai, China), the working concentration that used in this study was 2 μm.

### Western blotting

Western blotting analysis used in this study was performed as previously described [30]. The antibodies used were as follows: anti-HK2 (1:500 dilution, sc-374,091, Santa Cruz, USA), anti-GAPDH (1:100 dilution, sc-47,724, Santa Cruz, USA), anti-Vimentin (1:1000 dilution, sc-6260, Santa Cruz, USA), anti-ZEB1 (1:500 dilution, sc-81,428, Santa Cruz, USA), anti-ZEB2 (1:500 dilution, sc-271,984, Santa Cruz, USA), anti-MMP9 (1:1000 dilution, sc-21,733, Santa Cruz, USA), anti-CDH2 (1:500 dilution, sc-59,987, Santa Cruz, USA), anti-Fibronectin (1:500 dilution, sc-8422, Santa Cruz, USA), anti-p27 (1500 dilution, sc-1641, Santa Cruz, USA), anti-p21 (1500 dilution, sc-6246, Santa Cruz, USA). The horseradish peroxidase-conjugated anti-rabbit or anti-mouse IgG was purchased from Thermo Fisher Scientific (New York, NY, USA). GAPDH was used as the control and for quantification.

### Cell growth assays

Cell proliferation was detected by cell growth curves: 3 × 10^4^ cells were seeded in 6-well plates in triplicate, and cell numbers were counted every 2 days by using hemocytometer.

Cell viability was assessed using 3-(4,5-dimethylthiazole-yl)- 2,5-diphenyl tetrazolium bromide (Sigma-Aldrich, St Louis, MO, USA) dye, and the absorbance value at 490 nm was detected by using plate reader.

### Flow cytometry analysis

FACS (BD Biosciences, San Jose, CA, USA) was performed for cell cycle analysis, and the data was analyzed by using the Cell-Quest software. 1 × 10^6^ HK2 modified cells were washed with cold PBS for twice, then fixed in cold ethanol (70%) at 4°Covernight. Next day, cells were washed with cold PBS for twice, then treated with RNaseA (Sigma-Aldrich, St. Louis, MO, USA) and stained with propidium iodide (Sigma-Aldrich, St. Louis, MO, USA).

### In vitro migration and invasion assays

For the wound-healing assay in vitro, cervical cancer cells stably transduced with HK2 were plated in 6-well plates and cultured in DMEM containing 10% FBS. When the cells grew to nearly 100% confluence, the cell monolayers were scratched by using a pipette tip and washed with PBS and then cultured in DMEM without FBS at 37 °C. Then, cell monolayers were photographed by using a digital camera mounted on an inverted microscope at 0, 24 h and 48 h. The wound area was measured using ImageJ software, and the migration potential was calculated according to the equation: wound scratch area = (wound scratch area at 0 h) - (wound scratch area at 48 h or 72 h). Three independent experiments were performed for wound-healing assay.

For the migration and invasion assays in vitro, cells were added to upper transwell chambers with or without Matrigel (BD Biosciences, San Jose, CA) and incubated for 48 h. The medium in the upper chambers contained 1% FBS, and the medium in the lower chambers contained 10% FBS. The cells that migrated and invaded through the Matrigel-uncoated or -coated membranes were permeabilized with 70% methanol and stained with 0.1% crystal violet. Five fields for every chamber were photographed and counted by a scientist blinded to the experimental conditions. Three independent experiments were performed for the migration or invasion assays.

### Human tissue specimens and immunohistochemistry

A total of 25 human ovarian cancer tissues were collected from patients at the First Affiliated Hospital of Xi’an Jiaotong University during 2018 to 2020. These samples were all newly diagnosed, previously untreated. Detailed diagnostic and pathological reports were collected for all patients, and none of them had been previously treated with chemotherapy.

The Immunohistochemistry used in this study were performed as previously described [31]. The staining intensity was scored as: 0 (no staining), 1 (light brown), 2 (brown), 3 (dark brown). The percentage of positive cells was scored as: 0 = < 5%, 1 = 5 to 25%, 2 = 25 to 50%, 3 = 50 to 75%,4= > 75%. The immunohistochemistry (IHC) score = percentage score × intensity score. HK2 staining in tissues was classified into 2 categories (negative and positive expression): a score ≤ 1 was defined as negative, a score ≥ 2 was defined as strong positive. The antibodies used were as follows: anti-HK2 (1:200 dilution, sc-374,091, Santa Cruz, USA), anti-p-Akt1 (1:500 dilution, sc-293,125, Santa Cruz, USA), anti-MMP9 (1:300 dilution, sc-21,733, Santa Cruz, USA), anti-Fibronectin (1:500 dilution, sc-8422, Santa Cruz, USA).

### Real time PCR analysis

Total RNA extraction and the protocol for real-time PCR were performed as previously described. GAPDH was used as the housekeeping gene in this study, and all of the results were analyzed via the ∆∆Ct method. The primer sequences that used in this study for real time PCR were as follows: FN1 (F: 5-′CGGTGGCTGTCAGTCAAAG-3’R: 5-'AAACCTCGGCTTCCTCCATAA-3′), cyclin A1 (F: 5-'GAGGTCCCGATGCTTGTCAG -3’R: 5-'GTTAGCAGCCCTAGCACTGTC-3′), cyclin D1 (F: 5-'GCTGCGAAGTGGAAACCATC-3’R: 5-'CCTCCTTCTGCACACATTTGAA-3′), cyclin E1 (F: 5-'AAGGAGCGGGACACCATGA-3’R: 5-'ACGGTCACGTTTGCCTTCC-3′), p21 (F: 5-'TGTCCGTCAGAACCCATGC-3’R: 5-'AAAGTCGAAGTTCCATCGCTC-3′), p27 (F: 5-'AACGTGCGAGTGTCTAACGG-3’R: 5-'CCCTCTAGGGGTTTGTGATTCT-3′), HK2 (F: 5-'GAGCCACCACTCACCCTACT-3’R: 5-'CCAGGCATTCGGCAATGTG-3′), CDH2 (F: 5-’ TCAGGCGTCTGTAGAGGCTT-3’R: 5-’ ATGCACATCCTTCGATAAGACTG-3′), ZEB1 (F: 5-’ GATGATGAATGCGAGTCAGATGC-3’R: 5-’ ACAGCAGTGTCTTGTTGTTGT-3′), ZEB2 (F: 5-’ CAAGAGGCGCAAACAAGCC-3’R: 5-'GGTTGGCAATACCGTCATCC-3′), Vimentin (F: 5-’ GACGCCATCAACACCGAGTT-3’R: 5-'CTTTGTCGTTGGTTAGCTGGT-3′), MMP9 (F: 5-’ TGTACCGCTATGGTTACACTCG-3’R: 5-'GGCAGGGACAGTTGCTTCT-3′), GAPDH (F:5-'CACCGTCAAGGCTGAGAAC-3′ and 5-'TGGTGAAGACGCCAGTGGA-3′).

### Statistical analysis

All statistical analysis in this study was performed with Graphpad Prism 8.0 software and SPSS software version 19.0. Two-tailed unpaired Student’s t-test was used to determine the statistical significance for 2-group analyses, and presented as mean ± SD. Post hoc test was performed for comparison among groups. Chi-square test was used for count data. In all of the tests, statistical significance was defined as *p < 0.05, **p < 0.01, ***p < 0.001.

## Data Availability

The datasets used and/or analysed during the current study are available from the corresponding author on reasonable request.

## Abbreviations

HK2: Hexokinases 2
EMT: Epithelial-mesenchymal transition
MMP9: Matrix metalloproteinase-9
ZEB1: Zinc finger E-box binding homeobox 1
ZEB2: Zinc finger E-box binding homeobox 2
OV: Ovarian serous cystadenocarcinoma
PI3K: Phosphoinositide 3-kinase
IRS: Immunoreactive score
CESC: Cervical squamous cell carcinoma and endocervical adenocarcinoma
KICH: Kidney Chromophobe
SARC: Sarcoma
PCPG: Pheochromocytoma and paraganglioma
LIHC: Liver hepatocellular carcinoma
LUAD: Lung adenocarcinoma
LGG: Brain lower grade glioma
DMEM: Dulbecco’s modified Eagle’s medium
IHC: Immunohistochemistry
SFP: Specific pathogen-free
